# Strategies for Prevention of Surgical Site Infection in Patients With CIED Implantation: A Literature Review

**DOI:** 10.7759/cureus.13021

**Published:** 2021-01-30

**Authors:** Strahil Vasilev

**Affiliations:** 1 Cardiology, Medical University of Sofia, Sofia, BGR

**Keywords:** cied, infection, prevention, antibacterial, implantation

## Abstract

In the last several years a stable increase in cardiovascular implantable electronic device (CIED) implantation has been seen, mainly because of the expanded indications for their usage. An important complication of CIED implantation is possible postprocedural infection (pocket or systemic), which is connected with high morbidity and mortality rates and carries a significant financial cost to the health systems. Although this complication is not that frequent (ranging from 1% to over 5% in different studies), it is associated with a significant burden to patients, clinicians, and the healthcare system and because of that attention should be placed on the prevention of the infection. In this article, we collected and summarized the information and data available, directly related to the problem of prevention of CIED infection. The results of different studies and guidelines, regarding prevention with antibiotics, antiseptics, and the usage of antibacterial envelopes are analyzed. Perspective is put on the new technology of using antibacterial envelopes for prevention of infections. The aim of this paper is to review the various infection prevention techniques and to highlight the most beneficial ones according to guidelines and worldwide studies.

## Introduction and background

Since October 8, 1958, when Senning and Elmqvist implanted the first definitive electronic pacemaker in Sweden, there have been amazing and fast developments in the field of electrophysiology [1]. Since those early implantations, there are millions of people with permanent pacemakers (PPM) and implantable cardioverter-defibrillators (ICDs), and it is clear that there is a significant increase in the number of patients receiving cardiovascular implantable electronic devices (CIEDs). Over the past several years, the numbers have been increasing even more; this is mainly because of the expanded indications for implantation, following the guidelines, and the results of large clinical trials. According to Greenspon et al., there was a 96% overall increase in incidences of CIED implantation between 1993 and 2008 and an average increase of 4.7% annually. During this period pacemaker implantation increased by 45% and ICD implantation, which had a rate of 6.1% per 100,000 persons in 1993, but in 2006 showed a rate of 46.2 per 100,000 persons of population, which is an amazing increase of 504% [2]. Voigt et al. also claimed that there is a significant increase in CIED implantation and they report an increase in the number of cardiac resynchronization therapy (CRT) implants from 0% between 1996 and 2002 to 14.3% in 2006, the continued increase represents a 12% increment [3]. Although these devices are life-saving for some patients and significantly improve the lives of others, they also expose the patient to a risk of potential complications. Keeping in mind the fact that many CIEDs are implanted in elderly people and people with comorbidities, these factors predispose to infection and other complications. Infections are causing an increase in the morbidity and mortality rates in CIED patients. They often require complete removal of the device and are associated with a significant financial cost and burden to the worldwide health system [2-5].

## Review

CIED infection

CIED implantation is an invasive procedure and like every other invasive procedure, infection is a possible complication. Most CIED complications occur in-hospital or during the first six months after implantation [6]. Mechanical complications (coronary sinus dissection or perforation, pneumothorax, haemothorax, pericardial effusion or tamponade) are 3.2% of all complications, 6.2% are associated with lead problems and the least common complication, but the one with the highest morbidity and mortality is infection at 1.4% [6]. A frequent complication is haematoma formation, which can be treated more easily compared to the other complications, but can actually be a step towards infection. Haematomas take up 2.9-9.5% of the complications and no more than 2% of them will require evacuation. Even though haematoma formation is a minor complication, it results in prolonged hospital stay and hospital readmissions and it predisposes to infections [6-8]. Kirkfeldt et al. analyzed the complications after CIED implantation in Denmark and according to their results 9.5% of 5918 patients had at least one complication and 0.6% had more than one. They found that local pocket infection was seen in 22 (0.4%) of all patients and the risk of infection was lower in first implants, compared with generator replacement procedure. Lead-related reintervention was the most frequently seen complication, followed by haematoma with an occurrence of 2.3% in all patients. An interesting finding was that haematoma formation was seen more in underweight people than people with normal weight [8]. The rate of complications goes up as the center implantation volume decreases, low volume operators (<50 procedures annually) have a higher risk of complications [6,8]. According to Kirkfeldt et al., higher complication risk had centers with <750 procedures annually and operators with low volume had a higher risk of cardiac perforation and haematoma formation than higher volume operators [8]. A similar study done by Ranasinghe et al. [9], which included 81304 patients, shows that 6664 (8.2%) of all patients included had a major device-related complication and in 2710 (3.33%) of them, the complication occurred during their hospital stay. Again, the most common complication was lead reoperation. Mechanical complications (2.13%) and infections (0.73%) were the most common event for rehospitalization. Ranasinghe et al. report a great variation of the complications between different hospitals, too. Although it is clear that infections are not a common complication, they are complex for treatment, increase the length of stay, and are expensive to treat, costing $5,000 to $20,000. According to the literature available, treatment of CIED infection can be up to $83,000 per implant, which calculated to an average infection rate of 5.3% results in around $3.8 billion worldwide. For these reasons, more attention should be placed on prevention and exact treatment [8,9].

Pathogenesis of cardiac implantable electronic device infections

PPM infections are an important complication, which ranged between 0.13% and 19.9% in the early 1970s when first recognized. In the beginning, abdominal implantation was used, but when prepectoral implantation was invented, the incidences of infection went down to 7%. A long term follow-up of 959 patients showed that the infection rate of pectoral system implantation is 0.5% and abdominal is 3.2%. Even though lower incidence came with prepectoral implantation, the rate of CIED infections has been increasing [10,11].

Two major mechanisms can cause CIED infections. Contamination of the pulse generator or the leads, during implantation or following manipulations, is the most common cause. Bloodstream infection is the second mechanism that can cause CIED infection. Infection of the pulse generator causes so-called pocket infections. The intracardiac portion of the electrode can be infected through hematogenous bacteremia, but pocket infection can also spread along intravascular and intracardiac portions of the electrode and lead to their infection. Distant infectious foci (bacterial entry via the skin, urinary, gastrointestinal tract, osteomyelitis or thrombophlebitis) can cause direct contamination of the electrode leads. The factors, which play a role in the pathogenesis mechanism, are related to the device, microorganism, and the host.

Everything leading to impaired immune system of the host is a risk factor for infection of the CIED. Leading risk factors related to the host are renal dysfunction, diabetes mellitus, heart failure, and anticoagulant usage. At the time of the skin incision and device implantation, the patient’s own skin flora could be introduced in the wound and subsequently lead to pocket infection. This direct contamination via pocket erosion is a reason why stabilizing and fixating the pocket could contribute to primary prevention of CIED infections [5,10,11].

Devise-related factors affect the bacterial adherence to the leads or to the pulse generator. The type of the plastic polymer, hydrophobic surface, or irregularity of the device’s shape and surface are all factors that can predispose to infection. The more hydrophobicity and irregularity of the device, the greater the risk of adherence. An important factor is the polymer used, because polyethylene allows better adherence than polyurethane, while polytetrafluoroethylene allows less adherence than polyvinylchloride and silicone. The type of metal used is of importance, because titanium leads to less bacterial adherence than stainless steel. The best device is one with a smooth surface, less hydrophobic, made of titanium and with polyurethane or polytetrafluoroethylene [5,10,11].

*Staphylococcus aureus* (SA) is the most common bacterial organism involved in CIED infections. Even though there is a variation between countries and hospitals, methicillin-resistant staphylococci are the most frequently found bacterial species in CIED infections. A key step in the process of CIED infection is the attachment of the microorganism to the device and the surrounding tissue. Binding to the host is caused by adhesins that the microorganisms have on their surface. Microbial surface components reacting with adherence matrix molecules (MSCRAMMs) is the collective name of these surface adhesins, which can bind to fibrinogen, fibronectin, and collagen, which coat the surface of the CIED [10]. As said above, SA is the most common microorganisms found in CIED infections. SA is a coagulase-positive, Gram-positive, nonmotile, coccoid bacterium found in the human commensal microbiota. Methicillin-resistant *S. aureus* (MRSA) was first described in England in 1961. MRSA originated in healthcare settings and in communities and because of that is the leading microorganism causing hospital-acquired infections [12,13]. Attention should be placed on the formation of biofilm on CIEDs, which can predispose to infection. Biofilm forms in more than 25% of the implanted devices and it is known that these biofilms are a protective environment for microorganisms. This fact could prevent the effectiveness of short-term system antibiotics. There are some suggestions that local antiseptic infiltration of the device pocket and local perioperative antibiotic prophylaxis could reduce the biofilm formation. Steps towards understanding how to prevent biofilm formation are needed, because it will be helpful for better prevention of CIED infections since the bacteria in biofilms can remain inactive for years before clinical presentation and biofilms can reduce the antibiotic effects against the bacteria [14].

Results from Tarakji et al. [15] report that 78% of all infections in their study were monomicrobial and only 10% were polymicrobial. Aerobic gram-positive organisms accounted for 366 (88%) of the total 414 pathogens that were isolated. Out of all aerobic Gram-positive organisms, 331 (90%) were Staphylococci. Positive cultures could be taken from several places and the most common source according to Tarakji et al. was the lead tip (54%), but the pocket tissue is also a good source with 44%. The same percent (44%) was the rate of the methicillin resistant SA [15]. Although SA is the most common microorganism, a variety of coagulase-negative Staphylococcus (CoNS) have been recognized to cause CIED infections. Methicillin resistance is often seen in CoNS; one study reports that 53% of all CoNS were methicillin-resistant. Chua et al. [16], who studied 123 patients with CIED infections, stated that 81% of the pocket tissue cultures were positive. Out of all patients, 68% had an infection with CoNS and 24% had an infection with SA. In their study, 13% of the patients were infected with polymicrobial flora [11,15,16].

In summary, the factors which are associated with the greatest risk of developing a CIED infection are immunosuppression (renal dysfunction or corticosteroid usage) and predisposition to infections (diabetes mellitus), comorbidities of the patients, oral anticoagulant usage, operator experience, insufficient periprocedural prophylaxis and devise revision or replacement procedures [11].

Prevention of CIED infections

As said above, infection is one of the most worrying post-procedural complications to CIED implantation. Because of that the preparation and the initiation of the CIED implantation procedure should be carefully done, keeping in mind possible infection as a complication. The physician can do several things to minimize the chance of infection: carefully choosing and preparing the patient for the procedure, perioperative antibiotic prophylaxis, and antiseptic prophylaxis. Since there are few or no definitive guidelines for prevention of CIED infections, these steps are being used by many clinicians [3,6]. Choosing the right patient is not an easy decision, due to the aging population in the world and the expanded indications for CIED implantation. Young patients and those without comorbidities will have different peri- and postoperative periods than others and because of that, the way we cope with patients is individual. Overall, perioperative antibiotic prophylaxis is proven to benefit and to lower the incidences of CIED infections. According to Uslan et al., patients who received postoperative parenteral and postdischarge oral antibiotics had a slightly higher infection rate (1.4%) than patients who received only preincisional antibiotics (0.9%) [17]. Classen et al. report that intravenously antibiotic administration prior to the procedure is connected with lower infections. This finding, even though it is not exactly for device implantation, is again showing that administration of perioperative antibiotics is acceptable [18]. Risk reduction when using antibiotics one hour prior to the procedure is 40-95% compared with no antibiotics [19]. Postoperative antibiotics are not recommended because there is no data showing a benefit from it, as we saw from the study done by Uslan et al. [17]. On the other hand, Krahn et al. claimed that treatment with preprocedural antibiotics (cefazolin and vancomycin), intraprocedural bacitracin pocket wash, and two days post-procedural antibiotics (cefazolin) is beneficial and leads to lower infection rates, since the group that was treated according to this regimen had 0.78% infection rate compared to the other group receiving preprocedural antibiotic (cefazolin), of which only 1.03% had infection [20]. A very important part of prevention is the selection of the right antibiotic. The more commonly used ones are cefazolin, cefepime, ciprofloxacin, levofloxacin, linezolid, and vancomycin. A large meta-analysis done by Darouiche et al. showed that the most used regimen is based on one antibiotic, which is systematically administered via intravenous (IV) infusion one hour or less before CIED implantation. This meta-analysis shows that perioperative IV antibiotics lower the rate of infection compared to no antibiotics, and even more perioperative IV antibiotics showed a significant reduction of the incidences of infection compared to postoperative antibiotic administration [14]. 

Cleaning the patient’s skin around the site of device implantation in order to prevent surgical site infection is called antiseptic prophylaxis. The cleaning includes antiseptics like povidone-iodine and chlorhexidine. Darouiche et al. reported that according to their meta-analysis 0.5% alcoholic chlorhexidine is the most used antiseptic for prophylaxis of the surgical site [14]. Using povidone-iodine as an antiseptic is associated with higher infection rates than chlorhexidine gluconate. In the study by Uslan et al., 13 of all patients with CIED infection had their surgical site cleaned with povidone-iodine and only five of the patients with CIED infection were treated with chlorhexidine gluconate [17]. Information from a study comparing the two antiseptics also reports that chlorhexidine gluconate is associated with a significantly lower infection rate than povidone-iodine [21]. 

Haematoma formation is a minor complication, which is procedural related and is a base for the formation of a CIED pocket infection. Uslan et al. identified that patients with postoperative haematoma have a higher incidence of pocket infection than those without haematoma. With that said, postoperative haematoma is considered a risk factor for developing a CIED pocket infection and strategies for prevention should be applied. Achieving complete haemostasis in the pocket is recommended, by packing the pocket and providing tamponade, while placing the leads. There are some studies stating that topical thrombin application could be beneficial, especially for patients on anticoagulants. Although there is no or little data supporting these interventions, they are believed to be beneficial. Pressure dressing in the first 12-24 hours could be helpful for decreasing the risk of haematoma formation. Many patients needing CIED implantation will be on oral anticoagulants prior to the intervention. Anticoagulants are life-saving, but in this situation their effect is considered a risk factor for haematoma formation and subsequent infection. Because of this, interruption of the oral anticoagulants in patients with low risk of thrombo-embolic events could be recommended. Implantation of the CIED with a therapeutic international normalized ratio (INR) is acceptable, in order to avoid haematoma formation. Postoperative heparin usage is also associated with increased pocket haematoma and should be avoided [7,11,17,19].

In conclusion, we can say that according to the cardiology and surgical guidelines and the articles available, it is clear that a regimen of antibiotic IV administration an hour or less before the procedure and local surgical site cleaning with chlorhexidine gluconate is the combination that shows the greatest risk reduction. Even though this regimen's effectiveness is proven, there are a lot of unanswered questions, like exactly which antibiotic is the best choice for the different patients, how exactly to cope with anticoagulated patients, and more procedure-related techniques we can use in order to further minimize the CIEDs infections rate. 

Antibacterial envelope for CIED infection prevention

Although antibiotics and antiseptic prophylaxis are beneficial in CIED infection prevention, this complication is still a problem for clinicians and patients because of the high mortality and morbidity that comes with it. A few years ago, a new technology particularly concentrated on prevention of CIED pocket infection was introduced: the antibacterial envelope. This antibacterial envelope is basically an absorbable sterile prosthesis that creates a stable environment for the pulse generator when implanted in the body. It is an antibacterial multifilament knitted mesh that holds the CIED in place. Important to know is that this mesh is coated with an absorbable polyarylate polymer that acts as a carrier for antimicrobial agents. The absorbable antibacterial envelope holds the CIED in place and locally releases rifampin and minocycline (8 mg rifampin for medium-sized PPM and 11.9 mg for large PPM, 5.1 mg and 7.6 mg minocycline, respectively). The antibacterial envelope is absorbed for approximately nine weeks and the release of the antimicrobial agents directly into the CIED pocket is for a minimum of seven days. The antimicrobial activity of the envelope is directed against methicillin-sensitive SA, MRSA, *Staphylococcus epidermidis* and *S. lugdunensis*, *Acinetobacter baumannii*, and *Escherichia coli* [22]. The first generation of the antibacterial envelope was non-absorbable, but data still showed lower infection rates when using it. Although the antibacterial envelope releases antimicrobial agents directly in the pocket, at least one standard method of antimicrobial prophylaxis should be done. The most benefit from the usage of this technology is when in addition to the antibacterial envelope an oral or IV antimicrobial prophylaxis is used [22,23]. Another study by Bloom et al. reports a 99% success rate in antibacterial envelope implantation. The incidences of initial infection according to their study were 0% and in the procedures for replacement or revision were only 0.71%. The results of the study demonstrate a high rate of successful CIED implantation with antibacterial envelope and a low risk of infection [23]. One randomized controlled clinical trial, by Tarakji et al., studied the effects and safety of an absorbable, antibiotic-eluting envelope for the prevention of CIED pocket infection. The group that received the envelope consisted of 3371 patients, but in 10 patients implantation was not successful, so they report that the success rate of implantation was 99.7%. The reason for these 10 unsuccessful implantations was the limited pocket space. Regardless, CIED infections occurred in 32 patients in the envelope group and in 51 patients in the control (without envelope) group through the entire follow-up period of 12 months. There was a difference in the number of deaths between the two groups: 365 deaths in the control and 349 in the envelope group [24]. The antibacterial envelope is presenting a new way of preventing CIED pocket infections, but more data on the exact indications for its usage and the pros and cons in the long term should be collected and analyzed. 

## Conclusions

CIED infection complication carries high morbidity, mortality and financial burden. Prevention is a key step in lowering CIED infection rates. This can best be achieved by perioperative IV antibiotics an hour or less before the procedure and antiseptic prophylaxis with chlorhexidine gluconate. This regimen is proven to be beneficial in numerous studies, compared to intervention without perioperative IV antibiotic prophylaxis. In the last couple of years, a new method based on using antibacterial envelope has arisen and is showing promising initial results. However, further research is needed to clearly prove the benefit of these prevention techniques and to develop distinct guidelines for prevention of CIED infection.

## References

[1] van Hemel NM, van der Wall EE (2008). 8 October 1958, D Day for the implantable pacemaker. Neth Heart J.

[2] Voigt A, Shalaby A, Saba S (2010). Continued rise in rates of cardiovascular implantable electronic device infections in the United States: temporal trends and causative insights. Pacing Clin Electrophysiol.

[3] Greenspon AJ, Patel JD, Lau E (2011). 16-year trends in the infection burden for pacemakers and implantable cardioverter-defibrillators in the United States 1993 to 2008. J Am Coll Cardiol.

[4] Bongiorni MG, Marinskis G, Lip GY, Svendsen JH, Dobreanu D, Blomström-Lundqvist C (2012). How European centres diagnose, treat, and prevent CIED infections: results of an European Heart Rhythm Association survey. Europace.

[5] Blomström-Lundqvist C, Traykov V, Erba PA (2020). European Heart Rhythm Association (EHRA) international consensus document on how to prevent, diagnose, and treat cardiac implantable electronic device infections-endorsed by the Heart Rhythm Society (HRS), the Asia Pacific Heart Rhythm Society (APHRS), the Latin American Heart Rhythm Society (LAHRS), International Society for Cardiovascular Infectious Diseases (ISCVID) and the European Society of Clinical Microbiology and Infectious Diseases (ESCMID) in collaboration with the European Association for Cardio-Thoracic Surgery (EACTS). Europace.

[6] Brignole M, Auricchio A, Baron-Esquivias G (2013). 2013 ESC Guidelines on cardiac pacing and cardiac resynchronization therapy: the Task Force on cardiac pacing and resynchronization therapy of the European Society of Cardiology (ESC). Developed in collaboration with the European Heart Rhythm Association (EHRA). Eur Heart J.

[7] Nof E, Epstein LM (2013). Complications of cardiac implants: handling device infections. Eur Heart J.

[8] Kirkfeldt RE, Johansen JB, Nohr EA, Jørgensen OD, Nielsen JC (2014). Complications after cardiac implantable electronic device implantations: an analysis of a complete, nationwide cohort in Denmark. Eur Heart J.

[9] Ranasinghe I, Labrosciano C, Horton D (2019). Institutional variation in quality of cardiovascular implantable electronic device implantation: a cohort study. Ann Intern Med.

[10] Nagpal A, Baddour LM, Sohail MR (2012). Microbiology and pathogenesis of cardiovascular implantable electronic device infections. Circ Arrhythm Electrophysiol.

[11] Baddour LM, Epstein AE, Erickson CC (2010). Update on cardiovascular implantable electronic device infections and their management: a scientific statement from the American Heart Association. Circulation.

[12] Lee AS, de Lencastre H, Garau J, Kluytmans J, Malhotra-Kumar S, Peschel A, Harbarth S (2018). Methicillin-resistant Staphylococcus aureus. Nat Rev Dis Primers.

[13] Gordon RJ, Lowy FD (2008). Pathogenesis of methicillin-resistant Staphylococcus aureus infection. Clin Infect Dis.

[14] Darouiche R, Mosier M, Voigt J (2012). Antibiotics and antiseptics to prevent infection in cardiac rhythm management device implantation surgery. Pacing Clin Electrophysiol.

[15] Tarakji KG, Chan EJ, Cantillon DJ (2010). Cardiac implantable electronic device infections: presentation, management, and patient outcomes. Heart Rhythm.

[16] Chua JD, Wilkoff BL, Lee I, Juratli N, Longworth DL, Gordon SM (2000). Diagnosis and management of infections involving implantable electrophysiologic cardiac devices. Ann Intern Med.

[17] Uslan DZ, Gleva MJ, Warren DK (2012). Cardiovascular implantable electronic device replacement infections and prevention: results from the REPLACE Registry. Pacing Clin Electrophysiol.

[18] Classen DC, Evans RS, Pestotnik SL, Horn SD, Menlove RL, Burke JP (1992). The timing of prophylactic administration of antibiotics and the risk of surgical-wound infection. N Engl J Med.

[19] Kusumoto FM, Schoenfeld MH, Wilkoff BL (2017). 2017 HRS expert consensus statement on cardiovascular implantable electronic device lead management and extraction. Heart Rhythm.

[20] Krahn AD, Longtin Y, Philippon F (2018). Prevention of Arrhythmia Device Infection Trial: The PADIT Trial. J Am Coll Cardiol.

[21] Darouiche RO, Wall MJ Jr, Itani KM (2010). Chlorhexidine-alcohol versus povidone-iodine for surgical-site antisepsis. N Engl J Med.

[22] Tarakji KG, Mittal S, Kennergren C (2016). Worldwide Randomized Antibiotic EnveloPe Infection PrevenTion Trial (WRAP-IT). Am Heart J.

[23] Bloom HL, Constantin L, Dan D (2011). Implantation success and infection in cardiovascular implantable electronic device procedures utilizing an antibacterial envelope. Pacing Clin Electrophysiol.

[24] Tarakji KG, Mittal S, Kennergren C (2019). Antibacterial envelope to prevent cardiac implantable device infection. N Engl J Med.

